# Analysis of Heterodimeric “Mutual Synergistic Folding”-Complexes

**DOI:** 10.3390/ijms20205136

**Published:** 2019-10-16

**Authors:** Anikó Mentes, Csaba Magyar, Erzsébet Fichó, István Simon

**Affiliations:** Institute of Enzymology, Research Centre for Natural Sciences, Hungarian Academy of Sciences, Magyar Tudósok krt. 2., H-1117 Budapest, Hungary; mentes.aniko@ttk.mta.hu (A.M.); magyar.csaba@ttk.mta.hu (C.M.); ficho.erzsebet@ttk.mta.hu (E.F.)

**Keywords:** dehydrons, inter-subunit interactions, intrinsically disordered proteins, ion-pairs, mutual synergistic folding, solvent accessible surface area, stabilization centers

## Abstract

Several intrinsically disordered proteins (IDPs) are capable to adopt stable structures without interacting with a folded partner. When the folding of all interacting partners happens at the same time, coupled with the interaction in a synergistic manner, the process is called Mutual Synergistic Folding (MSF). These complexes represent a discrete subset of IDPs. Recently, we collected information on their complexes and created the MFIB (Mutual Folding Induced by Binding) database. In a previous study, we compared homodimeric MSF complexes with homodimeric and monomeric globular proteins with similar amino acid sequence lengths. We concluded that MSF homodimers, compared to globular homodimeric proteins, have a greater solvent accessible main-chain surface area on the contact surface of the subunits, which becomes buried during dimerization. The main driving force of the folding is the mutual shielding of the water-accessible backbones, but the formation of further intermolecular interactions can also be relevant. In this paper, we will report analyses of heterodimeric MSF complexes. Our results indicate that the amino acid composition of the heterodimeric MSF monomer subunits slightly diverges from globular monomer proteins, while after dimerization, the amino acid composition of the overall MSF complexes becomes more similar to overall amino acid compositions of globular complexes. We found that inter-subunit interactions are strengthened, and additionally to the shielding of the solvent accessible backbone, other factors might play an important role in the stabilization of the heterodimeric structures, likewise energy gain resulting from the interaction of the two subunits with different amino acid compositions. We suggest that the shielding of the β-sheet backbones and the formation of a buried structural core along with the general strengthening of inter-subunit interactions together could be the driving forces of MSF protein structural ordering upon dimerization.

## 1. Introduction

Mutual synergistic folding (MSF) complexes are a unique subset of intrinsically disordered proteins (IDPs). MSF IDPs can adopt a stable structure during the interaction, without a pre-existing folded partner [1,2,3,4]. At the time of the mutual synergistic folding process, the participating IDPs of these complexes synergistically fold into a stable, globular complex. Demarest et al. (2002) investigated the first MSF interaction between the p160 transcriptional coactivator protein ACTR and the tumor suppressor CBP proteins. They found that this MSF complex contains many hydrophobic side-chains and highly specific intermolecular hydrogen bonds, as well as buried intermolecular salt bridges, which help to fold the complex [5]. Since IDPs often have a high net charge, and they have a small content of hydrophobic residues, they are usually not able to form a hydrophobic core [6]. However, MSF complexes contain more hydrophobic residues, presenting an exception to a general view of IDPs [7,8].

While IDPs mostly have low sequence complexity, MSF complexes are rather heterogeneous, like globular proteins. Furthermore, MSF proteinsare also heterogeneous in amino acid composition similar to globular proteins [8]. The residue-based disorder prediction methods, developed for identifying segments bound to folded proteins, cannot be used for detecting of MSF complexes. Systematic analyses are required to understand and predict these MSF interactions. Nevertheless, this is difficult to implement since a severe weakness of the literature is the little information available about these complexes. At present, the most comprehensive and systematic catalog of MSF complexes is the MFIB (Mutual Folding Induced by Binding) database containing 205 entries [9]. 

A protein in aqueous solution is only stable when it contains a hydrophobic core buried from water by polar residues. Furthermore, these polar residues shield most of the hydrophobic residues from the solvent. For the first criterion, the protein should contain more residues than a required minimum either as a monomer or as an oligomer. The fulfillment of the second criterion depends on the ratio of the polar and hydrophobic residues because the ratio of the surface and buried residues rapidly decreases by increasing the total number of residues. For a given hydrophilic/hydrophobic ratio, either a long polypeptide chain or oligomerization is needed. MSF proteins fulfill both criteria by oligomerization.

Recently, the physical background of homodimeric MSF complexes from MFIB [7] was analyzed. We identified the residues with solvent accessible main-chain patches (RSAMPs) and studied the “under-wrapped” hydrogen bonds (dehydrons), which are not shielded well enough from solvent [10]. Our results suggested that homodimeric MSF complexes contain more RSAMPs and dehydrons than homodimeric complexes where all the interacting chains are globular in their monomeric form. These properties should contribute to their disordered nature in monomeric form and to their folding in the oligomeric state. In this study, the role of this phenomenon for heterodimeric MSF complexes will be discussed. In the case of heterodimers, the interacting polypeptide chains have different amino acid compositions, which discriminates heterodimers from homodimers. The MFIB database contains, unfortunately, a much lower number of heterodimeric structures when compared to homodimeric ones. Furthermore, there are highly similar proteins among them, which makes redundancy filtering necessary. 

## 2. Results and Discussion

### 2.1. Sequence-based Analysis

**Figure 1:**
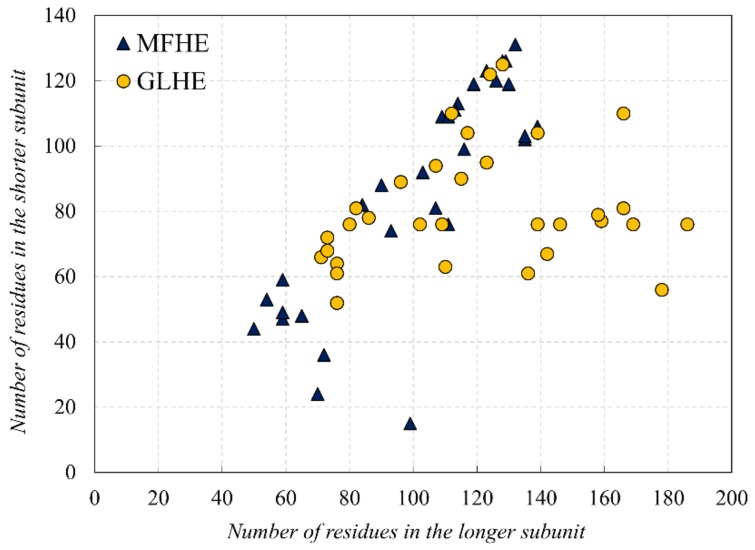
Comparison of the subunit lengths of the Mutual Synergistic Folding (MSF) (MFHE—blue triangles) and globular (globular heterodimeric GLHE—yellow dots) heterodimeric complexes.

In this study, first, we examined the amino acid composition of the MFIB heterodimeric (MFHE) complexes, which were compared with a globular heterodimeric reference dataset (GLHE), which has similar size distribution for the heterodimeric state (see Figure 1). Note that all GLHE subunits are more than 40 residues away from both axes, while the closest distance of an MFHE chain from the x-axis is less than 20 residues. Also, we will show later (see Figure 5) that the smallest identified globular monomer has 35 residues. In some cases, heterodimeric MSF complexes do not have enough amino acids for creating a hydrophobic core, but in most cases, they have as many residues as globular proteins have, thus other factors might also be responsible for the disordered nature of MFHE proteins.

**Figure 2:**
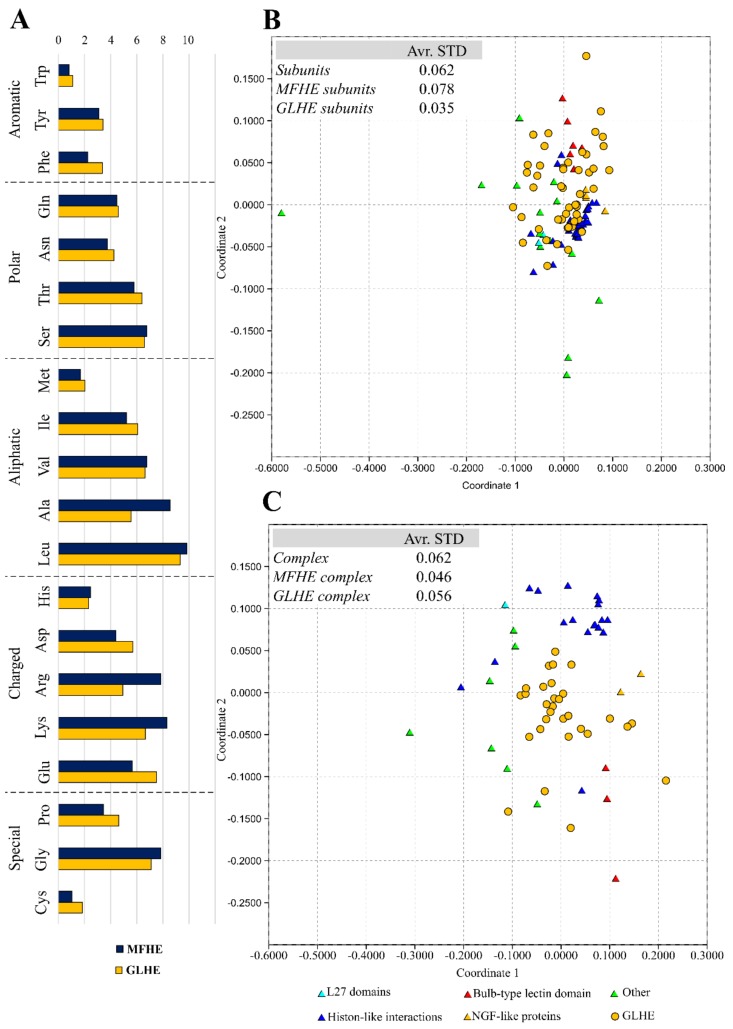
Amino acid composition of the heterodimer datasets, where the types of amino acids were grouped by Mészáros et al. [8] (**A**). The MFHE (triangles) and GLHE (dot) amino acid composition were compared using an indirect gradient analysis method, called Nonmetric Multidimensional Scaling (NMDS), which creates an ordination based on Bray-Curtis distances. In the plot, the objects are protein subunits (**B**) considered separately and complexes (**C**).

Since the beginning of the studies on IDPs, it is known they generally lack hydrophobic residues although alanine has a notably higher content in MFHE complexes compared to GLHE complexes, while the content of other aliphatic residues was similar among the two datasets (see Figure 2A). MFHE complexes have a high net charge, like non-MSF IDPs [11,12]. 

The amino acid composition of the MFHE and GLHE heterodimers was depicted by a rank-based, indirect gradient analysis method, called Nonmetric MultiDimensional Scaling (NMDS), which creates an ordination based on a distance or dissimilarity matrix, thus it allows decreasing a multidimensional and quantitative, semi-quantitative, qualitative, or mixed variables data set to two dimensions [13]. NMDS demonstrated a separation of MFHE and GLHE complexes and subunits (see Figure 2B,C). The amino acid composition of the subunits, whether globular or MSF complexes are formed, have equal distances from each other as the amino acid compositions of the complexes. Some differences are revealed between the two data sets—the NMDS of the amino acid composition of the MSF heterodimeric complexes showed smaller variation from the globular heterodimeric complexes (see Figure 2C), than the amino acid composition of the MSF subunits from the globular subunits (see Figure 2B). These differences can be explained by the fact that although the amino acid composition of the MSF subunits differs slightly from globular proteins, they are unable to fold into an ordered structure independently. The folding of an MSF subunit requires another partner, which in this case has a different amino acid composition, that could form MSF complexes which have similar amino acid composition than the globular subunits. NMDS also pointed out that MFHE is a diverse group based on their amino acid composition, and these complexes are also clustered according to their structural classes in MFIB [9].

The determination of the amino acids that contribute mainly to the observed difference was revealed by using SIMPER (similarity percentage) analyses. These amino acids were lysine (7.40%; 8.04%), alanine (7.30%; 7.90), leucine (7.14%; 6.64%), glycine (6.86%; 5.83%), arginine (6.39%; 6.70%), and glutamine (6.29%; 6.42%), which values support the similarity of the objects. Mostly aromatic and hydrophobic amino acids cause the amino acid compositions to separate (in slightly different proportions, See Table S1), which case is more common in heterodimeric MSF subunits and complexes if the MSF data were grouped via MFIB for comparison was considered, for the MFIB structural classes (see Figure 2, Table S1), with the exception of glutamine.

Most of the heterodimers from MFIB are histone-type proteins with their high content of lysine and arginine. Acetylated lysine and methylated arginine may interact with proteins containing bromodomains and Tudor domains within the disordered proteins that affect nucleic acid binding and RNA pathways [14]. 

The amino acid composition of the homodimeric complexes from MFIB (MFHO), heterodimeric MSF complexes was compared using our small globular protein (SGP) dataset as a standard reference by Kullback-Leibler divergence [15], which measures the extent of the dissimilarity between two probability distributions ($D=\underset{i}{\sum }p_{i}*\ln\frac{p_{i}}{q_{i}}$). MSF heterodimers show about the same similarity to MSF homodimers (D = 1.257) and small globular proteins (D = 1.879), while MSF homodimers are more similar to small globular proteins (D = 0.442). This result is in line with the observation that heterodimeric complexes from MFIB look much more disordered (~20%) than MFIB homodimers (MFHO) (~10%) [7] based on MoRFpred [16] and IUPred [17] results. Some regions of the heterodimeric MFIB complexes are also capable of folding on the surface of a globular protein. Most of these can be found in the DIBS (Disordered Binding Site) database [18]. It is rather rare, but it also shows the elevation of the group inhomogeneity. For example, the cellular tumor antigen p53 protein (UniProt: P04637) is able to establish a coactivator binding domain complex (MFIB: MF2201002, PDB: 2l14) with the CREB-binding MSF protein, although at the same part of the p53 capable to form a transactivator domain complex (DIBS: DI1000009, PDB: 2ly4) with the highly mobile folded B1 protein. We have also found examples of disordered proteins from UniProt (e.g., ID: Q9Y6Q9, Nuclear receptor coactivator 3) which are able to establish an MSF interaction (MF2201001, PDB: 1kbh), and another region is able to form a DIBS interaction (DI1000313, PDB: 3l3x), forming two different types of disordered protein complexes. 

It is interesting to note, that a few MFIB homodimers occur in DIBS as ordered interaction partners. For example, the dynein light chain (Tctex-type) protein (UniProt: Q94524), which is disordered in monomeric form based on MFIB (MFIB: MF2110016, PDB: 1ygt), while this homodimeric complex is the ordered part of a DIBS-interaction complex (Cytosolic dynein intermediate chain bound to Tctex-type dynein light chain, DIBS: DI2100002, PDB: 3fm7). An additional example of these multiple structure organizations is the homodimeric S100BEF-hand calcium-binding protein superfamily (MFIB: MF2100013, PDB: 1uwo), which is the ordered component of a DIBS-interaction (RSK1 bound to S100B dimer, DIBS: DI2000012, PDB: 5csf).

Besides the amino acid compositions, other sequential parameters also display differences between GLHE and MFHE. Based on cleverMachine [19] calculations (*p*-value < 0.0001: 56 scale of all 80) and grouped properties results, membrane proteins (*p*-value < 0.0001: 7 scale of 10), nucleic acid binding (*p*-value < 0.0001: 3 scale of 10), disorder propensity (*p*-value < 0.0001: 8 scale of 10), α-helix (*p*-value < 0.0001: 9 scale of 10), β-sheet (*p*-value < 0.0001: 9 scale of 10), aggregation (*p*-value < 0.0001: 8 scale of 10), burial propensity (*p*-value < 0.0001: 10 scale of 10), and hydrophobicity (*p*-value < 0.0001: 2 scale of 10) properties in MFHE are in general stronger than in globular heterodimers (Reference number of the dataset: 196154). While there is no significant difference between the sequences of MFIB homodimers and globular homodimers (GLHO) in most of the properties (exception of some membrane proteins and aggregation scales; *p*-value < 0.0001: 8 scale of all 80) (Reference number of the dataset: 199533).

We analyzed the Pfam database in conjunction with the intermolecular stabilization centers (SCs, see Chapter 2.2. Structure-based analysis) [20] on MFIB heterodimeric and globular heterodimeric complexes (for detailed results, see Table S2). In the MFHE have found 59 Pfam domains in a total of 19 families, while the GLHE have 64 Pfam domains in a total of 37 families. In the case of globular heterodimers, 3 of the 30 complexes have interactions and SCs between the Pfam domains of the monomers, whereas, for MFIB heterodimers much more, at least 15 of the complexes have Pfam domains in which monomers interactions and intermolecular SCs were found. This result confirms that the folding of the MSF proteins is related to their functional role since, in many cases, the two subunits form the biologically relevant unit.

### 2.2. Structure-based Analysis

In our recent analysis of MSF homodimeric proteins, we found differences in several structural parameters between our dataset and a globular reference dataset. These structural features were investigated including solvent accessibility, hydrogen bonds, stabilization center content, and ion-pairs with an additional investigation of the buried structural core size. 

The inter-subunit interface was identified based on the solvent accessible surface area (SASA) calculations. However, an MSF protein subunit in itself does not have an ordered structure, structural properties were also calculated for their monomeric forms, which were created by deleting a polypeptide chain from the heterodimeric PDB structures. This is referred to as their “monomeric structure” hereafter. The all-atom SASA values were calculated for all residues from the heterodimeric and monomeric structures. If the dimeric SASA value was below 20% of the monomeric value, the residue was identified as an interface residue. In the case of the MFIB heterodimeric dataset, 908 interface residues were identified out of the 4615 residues, that is 19.7% of all residues participate in the formation of the interface. In the globular reference heterodimeric dataset 470 interface residues were identified out of the 5155 total residues, i.e., 9.1% of all residues are forming the interface. As a different measure of the interface region, all-atom SASA values were also compared. In MFHE, 27.3% of the total surface area becomes buried upon dimerization, while in GLHE, only 11.6%. This result is in agreement with the finding of Gunasekaran et al., that the per residue interface area is higher in disordered complexes [3] In MSF proteins, the larger interface contact area underlines the importance of inter-subunit interactions, thus inter-subunit interactions were considered hereafter.

**Figure 3:**
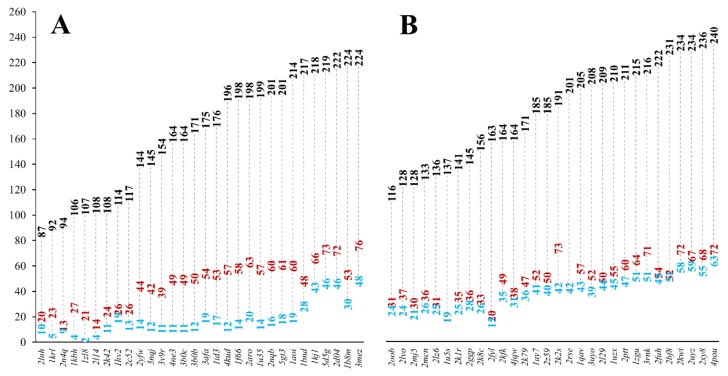
The number of burial residues in MFHE (**A**) and GLHE (**B**) complexes (black: number of all residues in a complex, red: number of buried residues in a heterodimeric complex, blue: sum of numbers of buried residues in the two monomeric subunits. See Figure S1 for the number of buried residues for the homodimeric MFHO and GLHO datasets.

Completely buried residues were identified in the MSF and the globular reference heterodimeric datasets using a stricter definition of burial, defining the core of the protein structure shielded from the solvent. We identified all residues, which have less than 10% relative all-atom solvent accessibility in the heterodimeric and monomeric structures, respectively. In MFHE, 10.8% of all residues are buried in monomeric form, while in GLHE this value is 20.9%. If the dimeric structures were analyzed, the values change to 27.7% and 26.3%, respectively. There are significantly fewer residues buried in the monomeric forms of MSF proteins when compared to globular ones. In the dimeric forms, the ratio of buried residues is similar in both cases. Figure 3 shows the number of buried residues in MSF (see Figure 3A) and globular heterodimeric complexes (see Figure 3B).

It can be seen that in the case of MSF heterodimers, there is a more considerable difference between the number of buried residues in the dimeric and monomeric forms, than in the case of globular heterodimers. In the case of globular heterodimers (see Figure 3B), the sum of the number of buried residues in the two monomeric subunits is close to the number of buried residues in the dimeric form. These subunits are ordered by themselves, and they do not need another subunit to help to order their structures. In the case of MSF heterodimers (see Figure 3A), the sum of the number of buried residues in the monomeric forms is lower than in the case of the globular heterodimers and, more importantly, they are much smaller than the number of buried residues in the dimeric form. These polypeptide chains are disordered by themselves, they need the presence of an interacting partner to help in ordering their structures. These protein chains need each other to form a reasonably sized core, needed for a stable, ordered structure.

The secondary structural element content was determined in the heterodimeric structures using the DSSP program [21]. We found that in the MFHE dataset, 43.6% of the residues have the α-helical conformation and only 16.1% of the residues belonged to β-sheets, in the globular heterodimeric dataset, these values were 21.5% and 27.5%, respectively. In the MSF, heterodimeric dataset β-sheets were less abundant than in globular heterodimeric proteins. This will have some consequences in the interpretation of our later results. 

We counted the number of inter-subunit ion-pairs. While there is only a small difference in the number of charged residues between MFHE and GLHE (1224 vs. 1380), the total charge is +320 for all 30 MFHE proteins and –91 for all 30 GLHE proteins. We found only 16 charged residues participating in 8 strong ion-pairs in the MFHE, while 28 residues are participating in 15 ion-pairs in the GLHE dataset. If we also consider weak ion-pairs, these values change to 73 residues participating in 42 ion-pairs for MFHE and 59 residues in 35 ion-pairs for GLHE. This is a 5.25-fold increase for MFHE and only a 2.33-fold increase for GLHE, respectively. Weak ion-pairs, presumably do not contribute to the enthalpic stabilization of the dimers, but probably play a role in the formation of electrostatic complementarity, already observed by Wong et al. in the case of complexes containing IDPs [22] This behavior was unexpected, and further investigation of the role of electrostatic interactions in the stabilization of MSF dimers is planned.

In the case of the MSF homodimers, we found that the main-chain solvent accessibility may play an important role in the stabilization of homodimer structures [8]. We identified residues with solvent accessible main-chain patches (RSAMPs). We have found a total of 161 RSAMPs in the MFHE dataset, and 90 RSAMPs in the GLHE dataset, respectively. There are 2 out of the 30 proteins in the MFHE dataset, which does not contain an RSAMP residue, while there are four such entries in the GLHE dataset. The average RSAMP content was 5.4 per heterodimeric complexes; thus, 17.7% of the interface residues are RSAMPs. In 26 of the 30 globular heterodimeric complexes, the average RSAMP content was 3, thus 19.1% of the interface residues are RSAMPs. 

**Figure 4:**
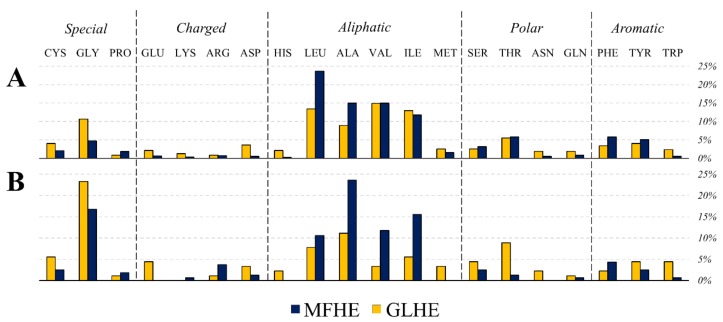
Amino acid composition of the interface (**A**) and the residues with solvent accessible main-chain patches (RSAMPs; (**B**)) of MFHE (blue) and GLHE (yellow) complexes.

On the one hand, the composition of the RSAMPs of MFIB heterodimers suggested that five types of amino acids (glycine, alanine, isoleucine, leucine, and valine) play a major role in these interactions (see Figure 4). These RSAMP contributing amino acids are mainly hydrophobic, are exposed to the inter-subunit interface. These residues do not contribute to the stabilization of the monomeric form since exposed hydrophobic surfaces are energetically not favorable. However, next to the favorable burial of their main-chain, they might help the formation of the tertiary structure by building sticky hydrophobic patches at the inter-subunit interface. On the other hand, in the case of the globular heterodimer dataset, the two amino acids with the smallest side-chains, glycine and alanine are the most abundant residues under RSAMPs. We investigated the secondary structural distribution of RSAMP, as well. We found that 33.5% of RSAMPs are located in β-sheets and 44.7% in α-helices. We checked the secondary structural composition of the interface residues, from which RSAMPs are selected. We found that 19.5% of interface residues have β-sheet and 63.9% have α-helical secondary structure. Considering the 3.3-fold higher occurrence of helical secondary structure at the interface, we can conclude that RSAMPs are more abundantly found in β structures, which can be easily broken by disturbing their hydrogen bonding network through interactions with accessible solvent molecules.

We counted the number of inter-subunit hydrogen bonds. We found a total number of 181 H-bonds in the MFHE and only 67 in the GLHE dataset, respectively. This is in agreement with our observation that inter-subunit interactions are of high importance in MSF heterodimers. We calculated the average wrapping of hydrogen bonds [10]. Hydrogen bonds with a low wrapping (dehydrons) are less shielded from the solvent. The average value was 13.8 for the MFHE and 14.6 for the GLHE. Inter-subunit hydrogen bonds are slightly less wrapped in the MSF heterodimers, which also indicates the importance of solvent accessibility.

We also identified inter-subunit stabilization centers in both the MFHE and GLHE datasets. Stabilization centers are special residue pairs, which together with their sequential neighbors, participate in above than average long-range interactions and are believed to contribute to the stabilization of protein structures [23]. The two residues that form a stabilization center are called stabilization center elements (SCEs). In MFHE, the average inter-subunit SCE content was 8.1, and we found at least one inter-subunit SC in 26 of the 30 heterodimers. In GLHE, the average SCE content was 0.5, and we found an inter-subunit SC is only 5 out of the 30 structures.

**Figure 5:**
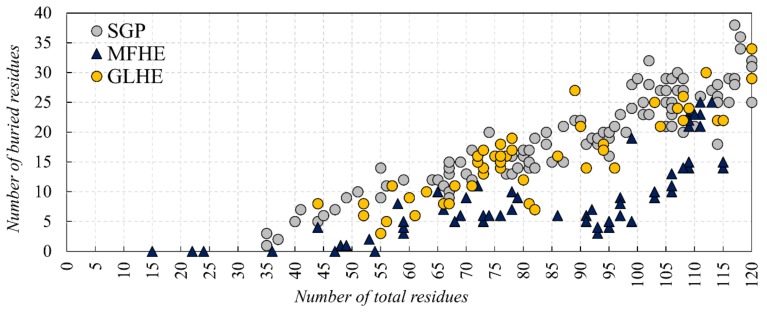
The number of total and buried residues of SGP (grey), GLHE (yellow) and MFHE (blue). For the number of total and buried residues of homodimeric MSF see Figure S2.

We investigated if there is a lower size limit for globular proteins, which already bear a buried core structure. Our analysis of monomeric, single-domain globular (SGP) dataset pointed out that proteins with 35 residues are already containing a buried structural core (see Figure 5). Our results, regarding the buried core size of the MFIB heterodimers, indicate that although a couple of polypeptide chains are too small to contain a buried core, this is not a general trend for the MFHE dataset. 

## 3. Conclusions

In our previous article [7], we found that the amino acid composition and sequence properties of MSF homodimers are similar to globular homodimers. However, they have more residues with solvent accessible peptide backbones that make them disordered in monomeric form, but they are ordered in a complex. There are some examples of these interactions in DIBS that prove their ordered nature. According to our results, MFIB heterodimers are less similar to globular proteins than homodimers, based on the calculated sequence and structural features. The MFIB heterodimers like the MFIB homodimers do not lack hydrophobic residues (as non-MSF IDPs), on the contrary, they are enriched in aliphatic residues which would theoretically allow the formation of a hydrophobic core, but in some cases, probably the chain itself is not large enough for the folding.

“Non-MSF” disorder prediction methods identify MFIB protein chains disordered at a short sequence segment which in some cases is confirmed by DIBS. In these DIBS interactions, heterodimeric MFIB subunits could bind to disordered, as well as globular protein regions. Therefore, in the case of heterodimeric MSF complexes, other factors can also affect their disorders than in the case of homodimeric MFIB proteins, because different factors are responsible for order-disordered interactions than for disordered-disordered complexes. This does not exclude that a protein chain can have the capability for both interactions and there has to be another ground why these proteins are unstructured on their own. In most cases of MSF heterodimers, the subunits themselves possibly do not have a low enough “energy” to fold, but the different compositions of the interacting partners may contribute to the stability of the complex. Understanding how sequence and composition and backbone variation affect foldability, will become increasingly crucial in folding protein design methods as more elements are included in the design process [24]. Based on NMDS results, the amino acid composition of the MSF heterodimeric complexes revealed smaller differences from the globular heterodimeric complexes, while the amino acid composition of the subunits showed distant similarity. Aromatic and hydrophobic amino acids are mainly responsible for the separation of the amino acid composition (based on SIMPER analysis) showed on NMDS. The amino acid composition of the MSF subunits is similar to globular proteins, but the MSF subunits together would change the amino acid composition of the complexes for a further reason. The heterodimeric MFIB complexes have a diverse amino acid composition, but they are involved in only a few types of molecular functions, such as DNA or histone binding (based on GO annotations from MFIB), which contributes to functional stability and making improvements in cell interactions [25].

In a recent paper [8], we concluded that MFIB proteins are disordered in monomeric form because they are too small to form a structural core. Our current analyses showed that however there are a couple of MSF protein subunits that do not contain buried residues, this is not a general rule, moreover we found that globular proteins with at least 35 residues already own a buried core (see Figure 5). At the same time, we found that the dimeric structures of MFIB and globular heterodimers contain a similar ratio of buried residues, but in monomeric form the MFIB heterodimers would contain only about half as much buried residues than globular heterodimers. We can conclude that the increased interface area of MFIB heterodimers contributes to the formation of a larger buried core structure. In globular heterodimers, the number of buried residues is increased only by a small margin upon dimerization, while in MFIB heterodimers there is a much larger increase (see Figure 3A). Globular monomers are stable and already own a reasonably sized buried structural core, while MFIB heterodimers are disordered by themselves and they need an interacting partner to form a large enough buried structural core to be stable. According to the structure-based analysis we can deduce that inter-subunit interactions are of high importance in the stabilization of MSF proteins. As in the case of homodimers, shielding of the main-chain from the solvent is an important factor for the stabilization of the heterodimeric structures. Interactions of the main-chain with water molecules might destabilize the secondary structure by breaking the hydrogen bond network, leading to the disruption of the secondary and the tertiary structure. Other interactions, which are identified by our definition of stabilization centers, play an important role in the stabilization of the heterodimeric structures, as well. This is consistent with our results about inter-subunit stabilization centers and Pfam domains, wherein the case of globular heterodimers, a few complexes have interactions and SCs between the Pfam domains of the subunits, whereas for MFIB heterodimers more than half of the complexes have inter-subunit interactions between the Pfam domains of the different chains. This also suggests that the folding of the MSF subunits is related to their functional role. 

Though we found that the difference in the number of RSAMPs between MSF and globular proteins is slightly smaller in the case of heterodimers than it was in the case of homodimers, considering the lower β-sheet content of the MFHE dataset, the RSAMP/β-sheet forming residue ratio is correspondingly high in MSF heterodimers and homodimers. We suggest that the shielding of the β-sheet backbones and the formation of a buried structural core together with the general strengthening of inter-subunit interactions together could be the driving forces of MSF protein structural ordering upon dimerization.

Protein folding, the structural organization of proteins in aqueous solution, is realized by monomolecular reactions of intermolecular interactions, even if this is followed later by further macromolecular interactions because of functional or stability reasons. In the case of MSF proteins for the formation of a stable ordered structure intermolecular interactions are needed, therefore it is part of the folding. Opposing the regular folding this is not a monomolecular, but rather a bimolecular reaction, in which the ratio of the participating components and other parameters can be changed. We believe that further experimental and theoretical investigation of the structural organization of MSF proteins can contribute to a more profound understanding of the folding problem.

## 4. Materials and Methods 

There are 49 heterodimeric proteins in the MFIB database. Entries belonging to the “coils and zippers” structural class were excluded, as in the case of homodimers. Since 25 of the 49 heterodimers are histones, filtering of the dataset was necessary to avoid overrepresentation and sequence redundancy of this protein class. Proteins were assigned to the same cluster if their sequence identity was over 90% using the BLASTClust toolkit 2.2.26 [26]. The 2mv7 entry was discarded because it was an outlier due to its fuzzy NMR structure in SASA calculations. One representative structure was kept for the remaining 30 clusters, creating the filtered MFHE dataset (see Table S3). A reference dataset was created of globular heterodimers (GLHE) from the PDBSelect [27] database with a total number of residues less than 240 to match the size distribution of the heterodimer MFIB dataset (see Table S3).

We described the methods in the latest article [7], but briefly: the interface term is used for the contact surface area of the two subunits in the heterodimeric structures. In cases where the term “monomeric structure” is used, calculations were carried out on single polypeptide chains, where the other chain was removed from the PDB files. Residues belonging to the interface region were identified based on solvent accessible surface area (SASA) calculations. All-atom SASA values were calculated using the FreeSASA 2.03 [28] program, residues where the SASA value calculated for the dimeric structure was less than or equal to 20% of the monomeric value, were defined to belong to the interface.

We were looking for residues in the interface that have solvent accessible spots in their main-chain in the monomeric structure, which become buried in the dimeric structures. We identified residues where the main-chain SASA in the dimeric form was less than 20% of the monomeric form value. Only residues with exposed main-chains, with a relative main-chain SASA larger than 0.2 in the monomeric structure, were taken into account. These residues with solvent accessible main-chain patches are called RSAMPs and are believed to be important for structural ordering upon dimerization of the disordered polypeptide chains collected in the MFIB database. 

We used an additional Small Globular Protein (SGP) dataset to determine the minimal buried core size of proteins (see Table S3). We collected monomeric single-domain proteins X-ray structures from the PDBSELECT database with less than 120 residues, which do not contain disulfide bonds. Since there was a significant hole in the size distribution of the X-ray structures, monomeric single-domain NMR structures without disulfide bonds were added to the dataset. We excluded rod-like and fuzzy NMR structures using a volume/surface cutoff criterion. Protein volumes were calculated using the ProteinVolume 1.3 program [29].

Secondary structural elements were identified using the DSSP [21] program. Hydrogen bonds were identified using the find_pairs PyMol command using 3.5 Å distance and 45-degree angle criteria [30]. Wrapping of hydrogen bonds was calculated using the dehydron_ter.py program [31]. Stabilization centers (SCs) are special pairs of residues involved in cooperative long-range interactions. The two residues that form a stabilization center are called stabilization center elements (SCEs). SCEs were identified using our SCide server [32]. Ion pairs were defined as pairs of positively and negatively charged residues, with a distance of less than a cutoff value between the charged groups. For strong ion pairs, this value is 4 Å [33], but we introduce additionally, a weak ion-pair definition with a distance cutoff value of 6 Å. Histidine residues were assumed to be neutral in these calculations because of the uncertainty of their protonation states. Ion pairs were identified using our own C++ program. We calculated the total charge of the proteins simply by adding the number of Arg and Lys residues and subtracted the sum of Asp and Glu resides.

Amino acid compositions were determined using MEGA7 software [34]. The amino acid composition of the protein subunits and complexes were visualized in Nonmetric multidimensional scaling (NMDS) in PAST3 [35]. In the plot, one point for each amino acid composition, where close points were more similar in composition (with Bray-Curtis distances). This was followed by a SIMPER analysis (also based on Bray-Curtis distances, in PAST3) to identify those amino acids that contributed most to the observed differences among the type of subunits and complexes. Disorder predictions were revealed by IUPred2A [17] and MoRFpred [16] algorithms.

## Supplementary Materials

Supplementary materials can be found at https://www.mdpi.com/1422-0067/20/20/5136/s1. Table S1. Contribution of the amino acids for the observed differences in NMDS by using SIMPER analysis in the subunits (A) and the complexes (B) Table S2. Pfam domains and the intermolecular SCs in globular (A) and MFIB (B) heterodimeric complexes. Table S3. List of PDB entries in the MFHE, GLHE and SGP datasets. Figure S1. The number of burial residues in MFHO (A) and GLHO (B) complexes (black: number of all residues in a complex, red: number of buried residues in a heterodimeric complex, blue: sum of number of buried residues in the two monomeric subunits. Figure S2. The number of total and buried residues of SGP, GLHE, GLHO, MFHE and MFHO.

## Abbreviations

IDP	Intrinsically Disordered Protein
MFIB	Mutual Folding Induced by Binding database
DIBS	Disordered Binding Site database
MFHO	MFIB Homodimeric dataset
MFHE	MFIB Heterodimeric dataset
GLHE	Globular Heterodimeric dataset
NMDS	Non Metric Multidimensional Scaling
RSAMPs	Residues with Solvent Accessible Main-chain Patches
SC/SCE	Stabilization centers/Stabilization center elements
SASA	Solvent Accessible Surface Area
SGP	Small Globular Protein dataset
