# Supplementary figures and images for: Analysis of Heterodimeric “Mutual Synergistic Folding”-Complexes
Source: Int J Mol Sci. 2019 Oct 16;20(20):5136. doi: 10.3390/ijms20205136 (PMC6829572; doi:10.3390/ijms20205136)

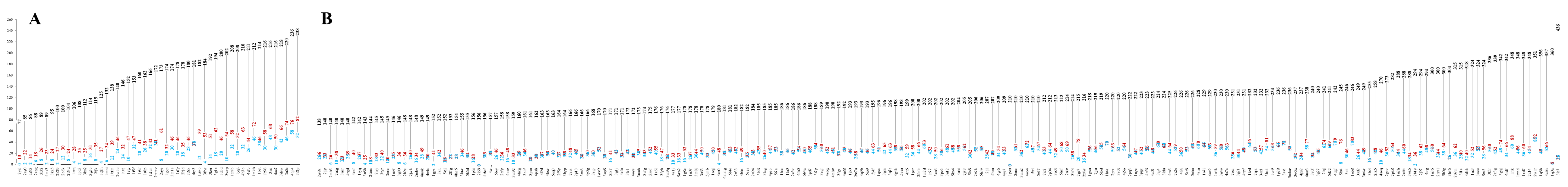

Supplement: Supplementary file 1 [file ijms-20-05136-s001.zip › Supplementary/FigS1.tif]

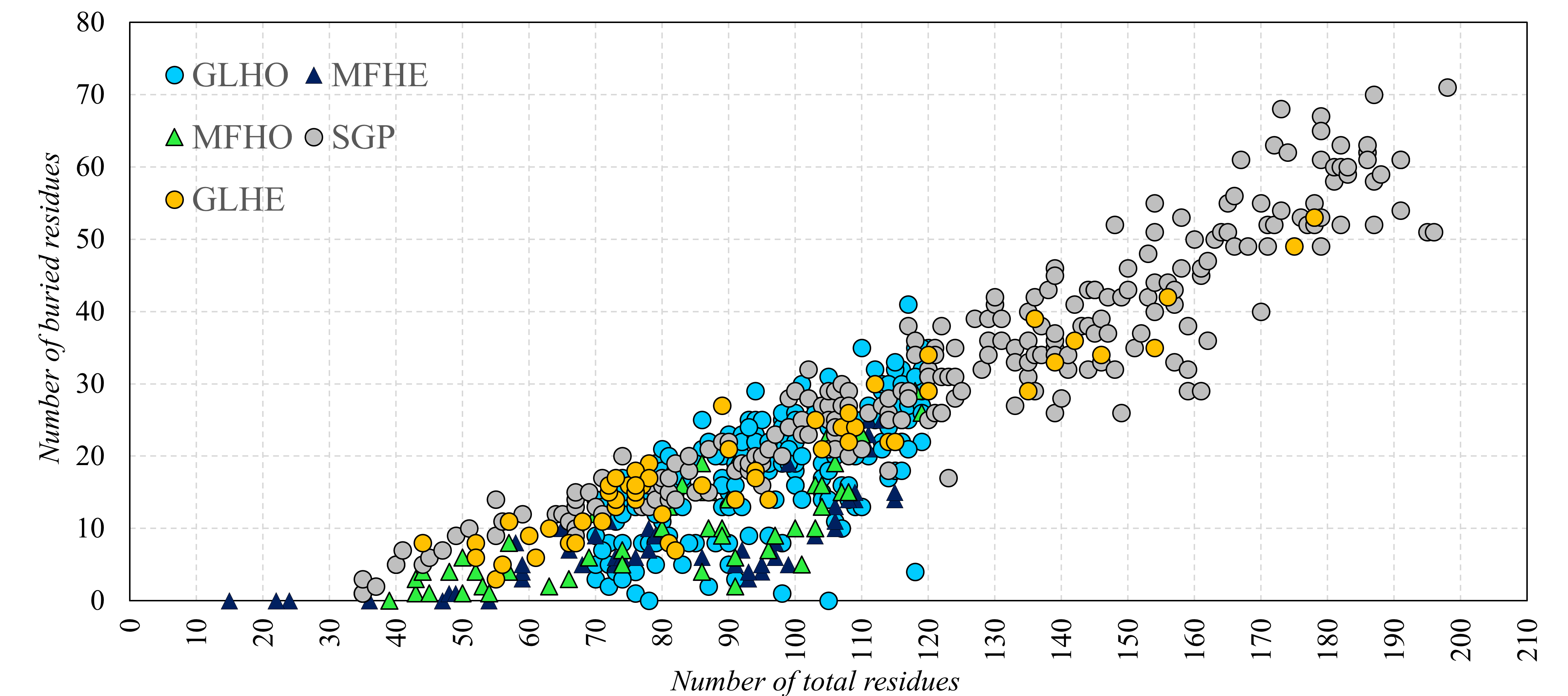

Supplement: Supplementary file 1 [file ijms-20-05136-s001.zip › Supplementary/FigS2.tif]
